# *Miconia papillosperma* (Melastomataceae, Miconieae): a new species from Amazonas, Brazil

**DOI:** 10.3897/phytokeys.63.7368

**Published:** 2016-05-13

**Authors:** Fabián A. Michelangeli, Renato Goldenberg

**Affiliations:** 1 Institute of Systematic Botany, The New York Botanical Garden. Bronx, NY 10458-5126, USA; 2 Universidade Federal do Paraná, Departamento de Botânica, Caixa Postal 19031, Curitiba, Paraná, 81531-970, Brazil

**Keywords:** Clidemia, seed morphology, taxonomy, Tococa

## Abstract

*Miconia papillosperma*, a new species of Melastomataceae shrubs from Northern Brazil is described and illustrated. This new species is characterized by elliptic lanceolate leaves with the only pair of secondary veins running close to the margin. It is also unique in having seeds with a papillose testa, a character until now unknown in the Miconieae. The description of this new species from a relatively well collected area near a major road north of Manaus, Amazonas, Brazil, is further evidence of our lack of knowledge on plants in many Neotropical areas.

## Introduction

As part of the NSF-funded project PBI-Miconieae (see http://sweetgum.nybg.org/melastomataceae), a large amount of unprocessed material and undetermined specimens have been seen by specialists in Melastomataceae in the last seven years, resulting on the determination of several thousand specimens at more than 20 different herbaria, the revision of several small and medium sized groups of this family (e. g. Judd and Ionta 2013; Judd et al. 2014; Gamba and Almeda 2014), and the description of almost 100 new neotropical species of Melastomataceae. During the course of the project’s herbarium work we found a group of specimens from Amazonas state in Brazil that merited further study. Upon close examination it became clear that these specimens belonged to a species of *Miconia* Ruiz & Pav. that had not been previously described.

*Miconia*, as traditionally defined by Cogniaux (1891), has almost 1100 species and is one of the largest genera of angiosperms, being also the largest exclusively Neotropical one (Goldenberg et al. 2013). In this traditional definition, *Miconia* is circumscribed as those members of the tribe Miconieae that have terminal inflorescences with flowers with rounded petals and that do not possess the diagnostic characters of other genera in the tribe that also have those characters (Goldenberg et al. 2013), such as *Calycogonium* DC., *Charianthus* D. Don, *Conostegia* D. Don, *Mecranium* Hook. f., *Pachyanthus* A. Rich., *Tetrazygia* Rich. ex DC., and *Tococa* Aubl. (see Skean 1993; Michelangeli 2000, 2005; Penneys and Judd 2005; Becquer Granados 2012; Judd et al. 2014; Kriebel et al. 2015).

With such a definition it is not surprising that molecular phylogenetic analyses have shown that *Miconia* is paraphyletic, with all other genera of the Miconieae nested within it (Michelangeli et al. 2004, 2008; Goldenberg et al. 2008). Due to this, some authors have advocated for an expanded *Miconia* that would encompass all the Miconieae (see Ionta et al. 2012). Since then, some species of Miconieae that would traditionally have been placed in other genera have been either recently described in *Miconia* (Ionta et al. 2012; Majure and Judd 2013; Michelangeli and Meier 2013; Gamba et al. 2014; Majure et al. 2014a, 2014b) or transferred to *Miconia* (Judd and Ionta 2013; Gamba and Almeda 2014; Judd et al. 2014).

The species described here has some superficial similarities with members of *Tococa* and *Clidemia* D. Don, and most collections that correspond to it have been determined as belonging to either one of those two genera. However, this new species does not fit the traditional definitions of either of these genera (sensu Cogniaux 1891), while it conforms to that of *Miconia*.

## Materials and methods

Herbaria in Brazil and the US (INPA, MO, NY, SP, RB, UPCB, US; acronyms following Thiers 2015) with important collections of Amazonian Melastomataceae were consulted in order to find specimens that could be assigned to this new species and in the search of putative relatives. Online databases were then queried to locate additional duplicates (http://www.splink.org.br/; http://sweetgum.nybg.org/vh; http://www.tropicos.org). All specimens listed were seen by at least one of the authors.

Seeds for Scanning electron microscopy were removed from a mature fruit, boiled in water for 5 min and the remnants of the fruit removed with forceps under light microscopy. The seeds were then mounted on aluminum stubs and sputter-coated with gold-palladium for 3 min in a HUMMER 6.2 Sputter Coater (Aratech LTD) and imaged on a JEOL ─ JSM 5410LV SEM at the NYBG structural botany laboratories.

Georeferenced data when available were taken directly from the specimen labels. Otherwise, specimens were georeferenced using the locality description and following those on Google Earth. For details of each specimen consult the NYBG virtual herbarium (http://sweetgum.nybg.org/vh).

## Taxonomy

### Miconia papillosperma

Taxon classificationPlantaeMyrtalesMelastomataceae

R. Goldenb. & Michelang. sp. nov.

urn:lsid:ipni.org:names:77154669-1

Figs 1 , 2

#### Diagnosis.

A shrubby species of berry-fruited Melastomataceae characterized by elliptic-lanceolate leaves with the only pair of secondary veins running close to the margin, and with abundant simple, red trichomes. It differs from morphologically similar species of *Clidemia* by the inflorescences that are terminal, and of *Leandra* Raddi by the flowers with rounded petals. It differs from morphologically similar species of *Tococa* by the lack of ant domatia and the seeds with the testa cells puzzle or S-shaped. It differs from all other Amazonian *Miconia* by the seeds with a papillose testa.

#### Type.

BRAZIL. Amazonas: Manaus–Caracaraí Road km 140, 1°48.09'S, 60°8.75'W, 27 Sep 1973, *C.C. Berg, F.A. Bisby, W.C. Steward, J.F. Ramos P18175* (holotype INPA; isotypes: K-000969284, MO-1726377, NY-01289602).

#### Description.

Shrubs 0.4–2 m. Young branches, petioles, primary, secondary and tertiary veins on abaxial leaf surface, adaxial leaf surface, hypanthium and calyx (for the latter two, see below) moderately to densely covered with red trichomes 1.5–5 mm long, filiform, unbranched, erect but sometimes with a curved apex (mostly on the leaves), sometimes gland-tipped (mostly on inflorescences and hypanthia, but sometimes on the leaves and branches too); upper portions of young branches and inflorescences, more precisely above the insertion of leaves/bracts usually with dense tufts of trichomes smaller and slenderer than the ones elsewhere on the plant, these trichomes 0.4–0.8 mm long; throughout the plant there are also sparse, short, reddish glandular projections, up to 0.1 mm long, elongate. Leaves opposite, equal to subequal in each pair, lacking ant-domatia; petioles 6–15 mm long; blades 3.8–13 × 1.5-4 cm, elliptic-lanceolate to elliptic, base narrowly rounded or broadly acute, apex acute to shortly acuminate (up to 3.5 mm long), margins hyaline, denticulate to crenulate, ciliate; nerves 3, basal, the outer pair 2.5–5 mm from the margin, (the marginal veins sometimes confluent at the base, i.e., joining the secondary veins instead of the primary), the axils of the secondary sometimes related with a deepened abaxial surface, suggesting mite domatia (but lacking membranes), the tertiaries more or less evenly spaced every 2.5–5 mm, the quaternaries very faint, nerves moderate to strongly impressed on adaxial surface, strongly prominent on abaxial surface. Inflorescences apical or seldom with an additional pair of inflorescences at the axils of the second leaf pair, 1.5–2.5 cm long (up to 3.5 cm long when fruiting), peduncled dichasia or short panicles with up to 2 (–3) pairs of paraclades, these simple or with compound dichasia. Bracts and bracteoles early caducous, 0.8–1.3 mm long, subulate, the margins with minute glands as described above, ending on a terminal trichome, usually glandular, 1.2–1.8 mm long. Pedicel 0.6–0.9 mm long. Hypanthium 3.1–4 × 3.2–3.7 mm, campanulate, terete (not costate), outside moderately (the surface of the hypanthium is visible) covered with glandular trichomes 2–3 mm long, erect or slightly curved, and also sparsely covered with the glandular projections described above, inside glabrous, torus glabrous. Calyx persistent, with the same indumentum as the hypanthium; tube 1.4 mm long; sepals 1.9–2.1 mm long, broadly triangular, apex rounded or obtuse, margins ciliolate; outer teeth 0.9–1.6 mm long, shortly subulate, slightly to clearly longer than the sepals. Petals white or pink, 6.1–7.6 × 4.3–5.5 mm, obovate, apex rounded to emarginate, margins dentate, and glabrous, except for one or two trichomes 0.5–0.9 mm long, glandular, erect, near the apex. Stamens 10, isomorphic, white; filaments 4.8–5.1 mm long, glabrous; connective not prolonged below the thecae, dorsally arcuate, with a minute dorsal tooth 0.1–0.2 mm long, acute; thecae slightly (0.1–0.2 mm) projected below the filament insertion, 3.9–4.5 mm long, slightly ventrally curved at the apex, this emarginate, pore apical. Ovary 2–3–locular, 2.7–3 mm long, ca. 1/3 inferior, conical, smooth (not costate), glabrous but with a crown of trichomes 1.2–1.6 mm long, erect, glandular; style 5.9–6.5 mm long, curved at the apex, glabrous, stigma 0.5–0.6 mm diam., truncate. Fruits 8–9.3 × 6–7 mm, vinose, urceolate. Seeds 1.3–1.4 × 0.9–1 × 0.7–0.8 mm, raphe ellipsoid, convex, hemi-ovoid in lateral view; testa papillose, the anticlinal walls puzzle or S-shaped.

**Figure 1.**
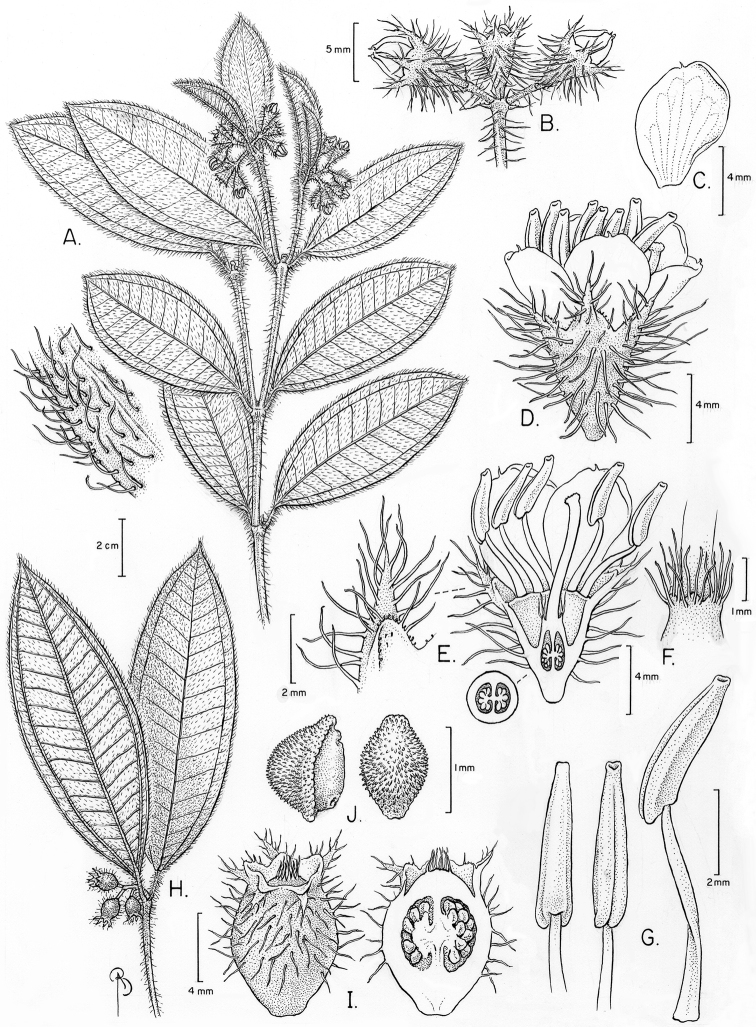
*Miconia papillosperma*. **A** Flowering branch **B** Detail of inflorescence **C** Petal, ventral view **D** Flower at anthesis **E** Longitudinal section of the flower showing hypanthia and ovary with details of an exterior calyx tooth and cross section of the ovary **F** Detail of the apex of the ovary **G** Stamens in dorsal, ventral and lateral view **H** Fruiting branch **I** Mature fruit in whole view and longitudinal section **J** Seeds in lateral and testa view. (**A–G** drawn from the NY isotype **H–J** from *Zaruchi 2564*, NY).

**Figure 2.**
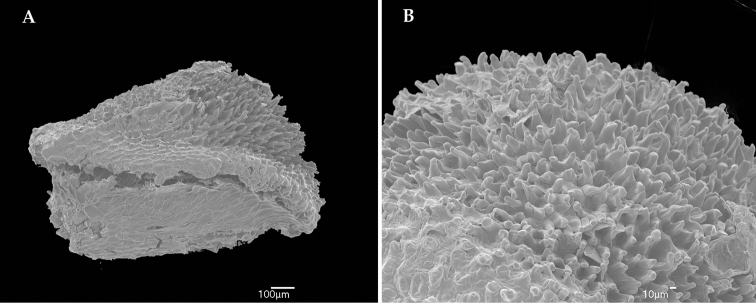
Scanning electron microphotograph of the seeds of *Miconia papillosperma*. **A** Whole seed **B** Detail of the papillose surface of the testa (from *Rocha 799*, NY).

#### Distribution and ecology.

**Figure 3.**
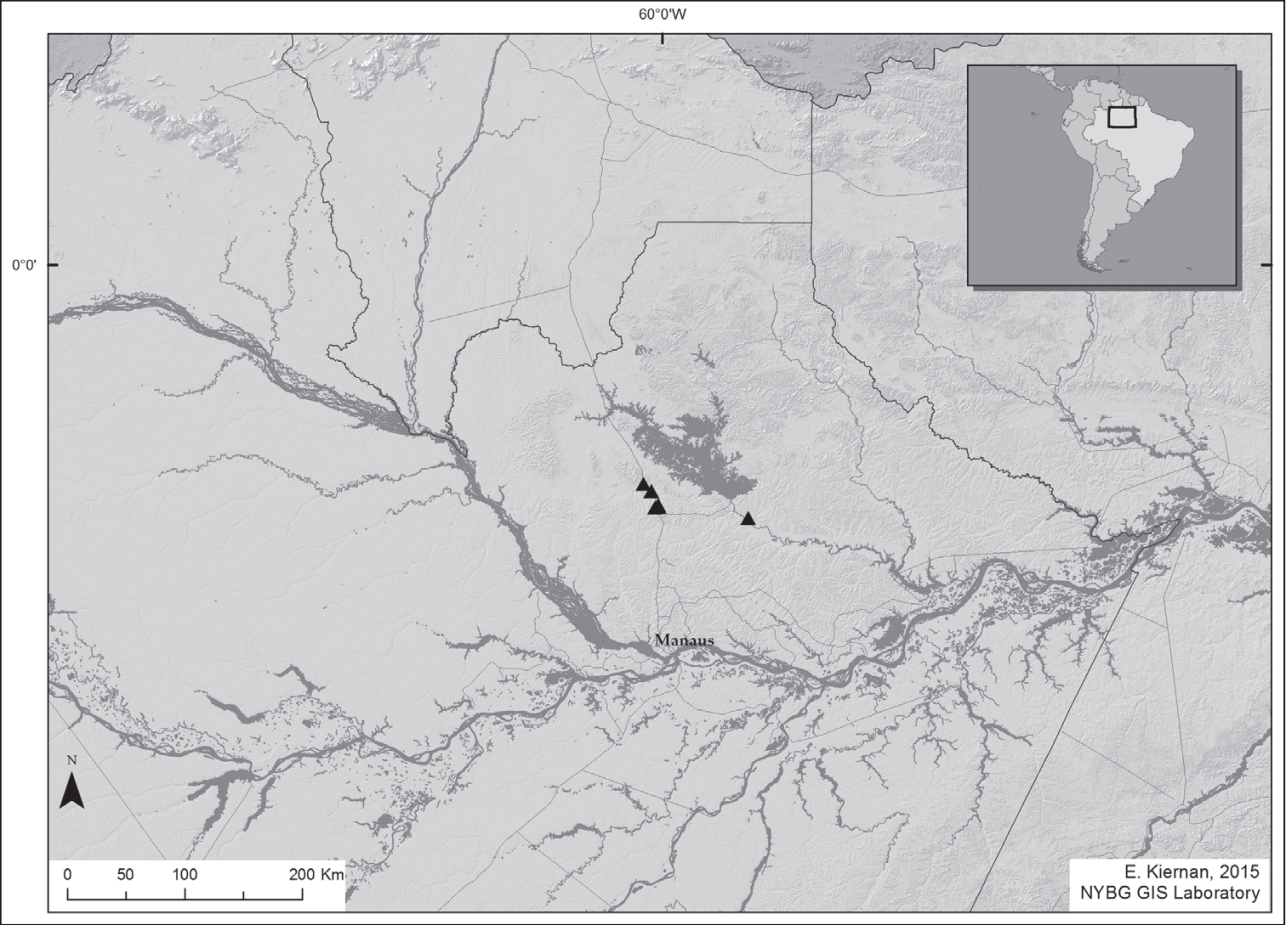
Geographic distribution of *Miconia papillosperma* (Amazonas, Brazil).

All specimens were collected along a 25 km stretch along the road between Manaus and Caracaraí (BR-174, from km 115 to km 140, north of Manaus), or in the vicinity of the city of Presidente Figueiredo, just outside the “Reserva Biológica do Uatumã”, about 75 km E (by air) of BR-174 . The plants grew on white-sand soil, associated to open vegetation locally recognized as “campina” (Fig. 3).

#### Phenology.

Collected with flowers and fruits from March to October.

#### Etymology.

The name reflects the unusual character of this species of having seeds with a papillose testa.

#### Conservation status.

The extent of occurrence (EOO) that includes both sets of localities is 768 km^2^ (Fig. 3). Even though some of the collections come from areas near the “Reserva Biológica do Uatumã” (a conservation unit kept by the Brazilian government), none have been made inside it. These are all areas near roads and with active low scale farming and logging. Following IUCN guidelines (IUCN 2012; IUCN Standards and Petitions Subcommittee 2014), we recommend that this species is categorized as endangered.

In the last five years at least six other Melastomataceae have been described from Amazonia (Goldenberg et al. 2011; Goldenberg and Meirelles 2011; Meirelles et al. 2015; Meirelles and Goldenberg 2014; Michelangeli 2014; Michelangeli and Goldenberg 2014). All but one of these species have very restricted distributions, underscoring that because our knowledge of plant diversity in Amazonia is still based mostly on a few well sampled localities, we still have a long way to go before we have a complete knowledge of the biodiversity of this region.

#### Additional specimens examined.

**Brazil. Amazonas**: Estrada Manaus–Caracaraí km 130, 1°51.57'S, 60°05.16'W, 10 May 1973, *B.W. Nelson et al. P21084* (INPA, MO, NY); Estrada Manaus–Caracaraí km 130, 25 May 1974, *W.A. Rodrigues et al. 9282* (INPA, UPCB); Estrada Manaus–Caracaraí km 130, 8 Aug 1974, *Artur (Loureiro) et al. s.n.* (INPA 43832); Estrada Manaus–Caracaraí km 115, 22 Sep 1977, *W.A. Rodrigues & M. Silva 9794* (INPA); Manaus–Caracaraí Road km 115, 1°58.86'S, 60°01.75'W, 14 Sep 1979, *J.L. Zarucchi et al. 2564* (INPA, NY, MO); Presidente Figueiredo, estrada Manaus–Caracaraí km 115, 9 Aug 1983, *C.A. Cid 4284* (INPA, K, NY, RB); Presidente Figueiredo, Campina das Pedras, ubicada en el Km 115 de la Rodovia BR-174 (Manaus–Caracaraí), en el lado oriental del Igarapé das Lajes, 29 Jun 985, *O. Huber 10665* (INPA, NY, SP); Presidente Figueiredo, Rebio Uatumã, Entorno, Estrada da Morena, ca. 40 km de Balbina, 21 Mar 2007, *J.G. Carvalho-Sobrinho 1439* (INPA, UPCB). Presidente Figueiredo, Rebio Uatumã, Entorno, Estrada assentamento, Ramal da Morena, 17 May 2007, *C.E. Zartman 7009* (INPA, UPCB); Presidente Figueiredo, Rebio Uatumã, Entorno, Estrada Balbina – Ramal da Morena, 2°4.88'S, 59°21.22'W, 26 Jul 2007, *J.E.L.S. Ribeiro 2863* (INPA, UPCB); Presidente Figueiredo, Cachoeira da Iracema, trilha da Cachoeira as margens do riacho, 1°59.33'S, 60°03.52'W, 10 Oct 2012, *M.J.R. Rocha et al. 799* (BHCB, NY).

#### Morphological comments.

*Miconia papillosperma* is a very distinctive species that really does not closely resemble any other species of Miconieae known to us. Most of the specimens are in fruit and this is presumably why it remained undescribed until now. The elliptic-lanceolate to elliptic leaves with the only pair of secondaries very close to the margin resembles some species of *Macairea* DC., an unrelated group with capsular fruits in the Marcetia clade (see Michelangeli 2013), but the leaf surface and indument are different. The shrubby habit and abundant red trichomes on the leaves and young stems resemble some species of *Clidemia*, *Leandra*, and *Miconia*, but no other species in these genera has the leaves of *Miconia papillosperma*. *Tococa rotundifolia* (Triana) Wurdack and *Tococa hirta* O’Berg ex Triana also have similar reddish trichomes, but in both species at least one of the leaves of each pair has ant domatia and the venation pattern is quite different, with the secondaries running near halfway between the primary vein and the margin and not towards the margins of the lamina (Michelangeli 2005). The flowers of *Tococa rotundifolia*, with a winged hypanthium, can’t be confused with those of *Miconia papillosperma*. However, the broad conical hypanthium with a calyx with subulate outer teeth, the anthers with a dorsal connective blunt tooth, and the ovary with a corona of glandular trichomes at the apex, does resemble other species of *Tococa*, most notably *Tococa ciliata* Triana and *Tococa hirta* (Michelangeli 2005). However, the seeds of all species of *Tococa* sensu stricto have the testa cells with straight walls (Michelangeli 2000; 2005), while the testa cells in *Miconia papillosperma* have clearly puzzle or S-shaped walls. In summary, we think that the best placement for this new species is within *Miconia* given its terminal inflorescence with flowers with rounded petals.

It should be noted that the seeds of *Miconia papillosperma* are unique within the tribe. To date the seeds of more than 500 species of the close to 2000 Miconieae have been imaged and studied and none of them have papillose testa (Groenendijk et al. 1996; Michelangeli 2000; Martin and Michelangeli 2008; Ocampo and Almeda 2013; Ocampo et al. 2014). Many species in different genera do have testas with convex or tuberculate cells, but these are not extended to form papillae. There is a clade within *Tococa* that has trichomes on the seeds, but these are morphologically different from the papillae of *Miconia papillosperma*, and they are on the raphe and not on the testa. The ecological and taxonomic significance of these papillae should be investigated further.

## Supplementary Material

XML Treatment for Miconia papillosperma
